# Fluid Flow to Electricity: Capturing Flow-Induced Vibrations with Micro-Electromechanical-System-Based Piezoelectric Energy Harvester

**DOI:** 10.3390/mi15050581

**Published:** 2024-04-27

**Authors:** Jin Gu Kang, Hyeukgyu Kim, Sangwoo Shin, Beom Seok Kim

**Affiliations:** 1 Department of Mechanical Engineering, Seoul National University of Science and Technology, Seoul 01811, Republic of Korea; nak9837@seoultech.ac.kr (J.G.K.); kimhyeuku@seoultech.ac.kr (H.K.); 2 Department of Mechanical and Aerospace, University at Buffalo, The State University of New York, Buffalo, NY 14260, USA; sangwoos@buffalo.edu; 3 Department of Mechanical and Automotive Engineering, Seoul National University of Science and Technology, Seoul 01811, Republic of Korea

**Keywords:** vibration-to-electricity conversion, MEMS energy harvester, fluid-induced vibration (FIV), Karman vortex, micro-electromechanical system (MEMS), piezoelectric film

## Abstract

We introduce a micro-electromechanical system (MEMS) energy harvester, designed for capturing flow energy. Moving beyond traditional vibration-based energy harvesting, our approach incorporates a cylindrical oscillator mounted on an MEMS chip, effectively harnessing wind energy through flow-induced vibration (FIV). A highlight of our research is the development of a comprehensive fabrication process, utilizing a 5.00 µm thick cantilever beam and piezoelectric film, optimized through advanced micromachining techniques. This process ensures the harvester’s alignment with theoretical predictions and enhances its operational efficiency. Our wind tunnel experiments confirmed the harvester’s capability to generate a notable electrical output, with a peak voltage of 2.56 mV at an 8.00 m/s wind speed. Furthermore, we observed a strong correlation between the experimentally measured voltage frequencies and the lift force frequency observed by CFD analysis, with dominant frequencies identified in the range of 830 Hz to 867 Hz, demonstrating the potential application in actual flow environments. By demonstrating the feasibility of efficient energy conversion from ambient wind, our research contributes to the development of sustainable energy solutions and low-power wireless electron devices.

## 1. Introduction

In today’s rapidly advancing technological landscape, the need for compact and efficient power sources has become increasingly critical [1,2,3]. This need is paramount not only in traditional arenas such as wireless sensor networks and wearable devices but also in rapidly growing fields like advanced mobility solutions and drone technology. These dynamic applications, characterized by their demand for autonomy and enduring power sustainability, are transforming our interconnected and mobile world. Specifically, the inherent movement of devices in sectors like drone technology and new mobility solutions introduces novel opportunities for energy harvesting [4,5,6]. This interaction with airflow represents an overlooked method to boost their sustainability and operational efficiency.

In this context, the development of micro-electromechanical system (MEMS)-based energy harvesters, which are capable of transforming kinetic energy from environmental forces like wind into electrical power, signifies a critical breakthrough [7,8]. Harvesters employing cantilever beams equipped with piezoelectric films excel in harnessing kinetic energy from diverse sources, including vibration, gravitational forces, and wind [9,10,11,12,13]. Additionally, the piezoelectric energy harvesting method, compared to electromagnetic and triboelectric energy harvesting methods, has the advantage of easier miniaturization through MEMS processes and is less affected by external conditions such as dust and humidity [14,15]. The materials commonly chosen for piezoelectric films include PZT and AlN. PZT, known for its high piezoelectric properties, has been applied in MEMS energy harvesters, typically harvesting energy ranging from 1 μW to 100 μW [16,17,18]. However, a significant drawback of PZT is that its use in micromachining processes can contaminate fabrication equipment. On the other hand, MEMS harvesters that employ AlN, while having lower piezoelectric properties compared to PZT, are noted for their ease of processing in MEMS fabrication. The energy harvesting capacity of AlN-based MEMS energy harvesters ranges from 0.8 nW to 10 μW [19,20]. However, the low power levels generated by energy harvesters pose challenges in electrical circuit operations such as rectification. Nonetheless, several studies have been conducted to rectify and boost power at the mV and nW-μW levels into usable scales for energy harvesting [21,22].

**Figure 1:**
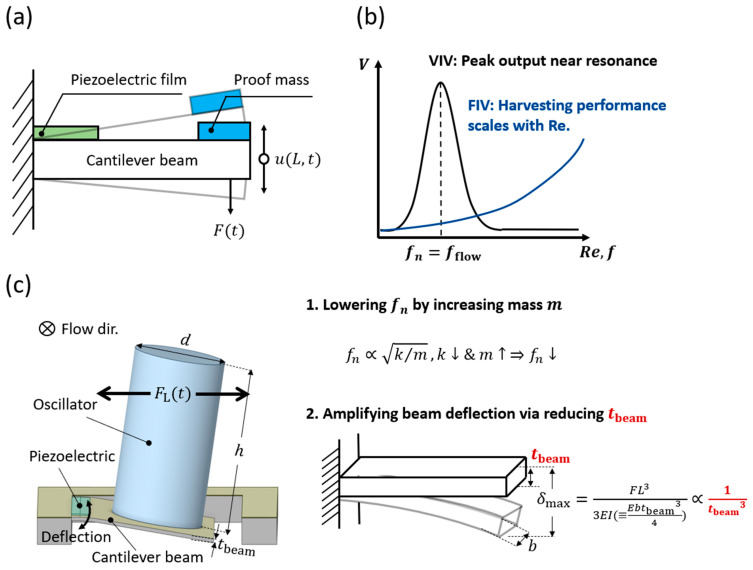
Schematics of energy harvesters: (**a**) Traditional MEMS-based vibration harvester consisting of a piezoelectric cantilever beam and a proof mass. (**b**) Conceptual comparison of operating regimes, where VIV-based harvesting peaks near resonance, whereas FIV-based harvesting seeks a sustained response as the Reynolds number increases. (**c**) Redesigned cantilever–oscillator harvester based on [29], retuned for high-Reynolds-number, off-resonant operation. The oscillator is subjected to an aerodynamic lift force acting perpendicular to the flow direction, which serves as primary time-varying excitation source for vibration. Increasing the oscillator mass m lowers the natural frequency $f_{n}$ , shifting it further below the flow-induced excitation frequency (upper-right inset). Reducing the beam thickness (t)  lowers the bending rigidity $\left(EI\right)$ , resulting in a larger deflection $\left(\delta \propto {t_{\mathrm{beam}}}^{-3}\right)$ and enhanced piezoelectric strain (lower-right inset).

Demonstrating a substantial energy density of approximately 0.3 μW/mm^3^, these devices are suitable for a broad spectrum of applications [10,23]. Conventional vibration energy harvesters, typically comprising a silicon cantilever beam with a piezoelectric film and a proof mass (Figure 1a), are noted for their manufacturing simplicity [23,24]. However, their efficiency predominantly relies on matching the external excitation frequency with their resonant frequency. To overcome the limitation of narrow operational bandwidths that is inherent in resonant systems, recent research efforts have been directed towards tweaking system parameters such as rigidity, operating frequency, and damping, thereby widening the bandwidth to adapt to varying environmental vibrations [25,26,27].

Building on these advances, flow-driven MEMS energy harvesting has also attracted growing attention. While vortex-induced-vibration-based harvesting concepts have been widely explored [28,29,30,31,32], most MEMS implementations have focused on resonance-based operation. In such designs, the response is maximized when the excitation frequency approaches the structural natural frequency (Figure 1b). However, resonance-based approaches inherently provide high performance only within a narrow frequency band, and the response can drop rapidly as operating conditions vary. Therefore, to achieve stable operation over a wide frequency range in realistic environments, a design principle that reduces reliance on resonance is needed. A representative application that demands wideband robustness is high-Reynolds-number flow. Using the Strouhal relation St=fD/U , the characteristic flow frequency is f=St U/D , indicating that for a fixed oscillator diameter D , f  increases with flow velocity ( U) . In the operating range considered here ( U≈8 m/s, D≈2 mm), the device has a first natural frequency of approximately 78 Hz [29], whereas the characteristic flow frequency under high-speed conditions ( U≥8 m/s) increases to approximately 840 Hz (Figure 1b), yielding a strong frequency mismatch ratio $f_{flow}/f_{n}\approx 10$ . Under such conditions, resonance-tuned VIV operation becomes practically constrained.

In this study, the device was redesigned to relax the dependence on strict resonance matching and to promote a more flow-governed harvesting response. As shown in Figure 1c, we adopted a cantilever–oscillator coupled configuration based on the cantilever–oscillator architecture [29]. In contrast to conventional designs that primarily rely on resonance matching, the present device was tailored for a high-Re operating condition in which the characteristic flow frequency is substantially higher than the structural natural frequency. To realize this operating regime, two structural modifications were introduced. First, the effective mass m of the oscillator was increased to reduce the natural frequency of the cantilever–oscillator system, following the scaling $f_{n}\propto \sqrt{k/m}$ . Thus, increasing m shifts the structural resonance further below the working flow-induced excitation frequency. Second, the cantilever thickness ( $t_{\mathrm{beam}}$ ) was reduced. For a rectangular cantilever, the area moment of inertia scales as $I\propto {t_{\mathrm{beam}}}^{3}$ , and the maximum deflection under a given load follows $\delta_{max}=FL^{3}/(3EI)\propto {t_{\mathrm{beam}}}^{-3}$ , indicating that the deflection increases strongly as the thickness decreases. In addition, since the bending stiffness scales as $k\propto EI/L^{3}\propto {t_{\mathrm{beam}}}^{3}$ , reducing $t_{\mathrm{beam}}$ also lowers the natural frequency. Therefore, increasing m and reducing $t_{\mathrm{beam}}$ both contribute to lowering $f_{n}$ , while the reduction in $t_{\mathrm{beam}}$ additionally promotes larger cantilever deflection under a given load. These two design features distinguish the present device from Ref. [29] and related prior work [28,29,30,31,32]. In the present design, the harvester is intentionally operated under a condition where the flow-induced excitation frequency is substantially higher than the structural natural frequency, while the thinner cantilever and heavier oscillator are used to enhance the beam deflection and further reduce $f_{n}$ . To validate this concept, we employ a combined numerical and experimental approach. Two-dimensional computational fluid dynamics (CFD) simulations are performed to analyze vortex formation and fluid–structure interactions, while wind-tunnel experiments are conducted to characterize the resulting flow-induced response of the fabricated device. Through this integrated methodology, we assess the feasibility of the proposed architecture for energy harvesting under high-speed airflow conditions.

This paper is organized as follows: Section 2 describes the structure and operating principles of the MEMS energy harvester, along with the methods and setups for numerical analysis and experiments. Section 3 investigates the behavioral characteristics of the MEMS energy harvester in wind environments through numerical analysis and explores the fabrication and properties of the harvester, demonstrating that actual electricity production aligns with the predicted characteristics through experimental validation. Finally, Section 4 discusses the conclusions of our research, future works, and areas for improvement.

## 2. Materials and Methods

In this section, we detail the design and fabrication of our MEMS energy harvester, numerical methods for quantifying the fluid forces, and experimental evaluation of the energy harvesting efficiency.

### 2.1. Design and Principle of MEMS Energy Harvester

Our study presents a MEMS energy harvester intended for flow environments, extending beyond the conventional resonance-centered design framework of vibration-based harvesters. The device is based on the cantilever–oscillator architecture reported in Ref. [29], but was redesigned for operation under relatively high-speed flow conditions, where strict frequency matching becomes less practical. In this regime, the characteristic frequency of aerodynamic forcing increases with flow velocity and tends to deviate substantially from the structural natural frequency. Under such conditions, the harvester is required to produce sufficient structural strain even under off-resonant excitation. To address this requirement, two physics-based structural modifications were introduced. First, the effective oscillator mass was increased while maintaining the projected area of the oscillator. This was achieved by replacing the hollow cylindrical oscillator with a solid cylinder of the same outer diameter. In this way, the projected area was preserved, while the inertial loading of the oscillator was increased. From the scaling relation $f_{n}\propto \sqrt{k/m}$ , an increase in the effective mass lowers the natural frequency of the cantilever–oscillator system. This modification was therefore intended to increase the frequency separation between the structural natural frequency ( $f_{n})$ and the flow-induced excitation frequency ( $f_{flow}$ ), thereby reducing the dependence of the device on strict resonance matching. Second, the cantilever thickness ( $t_{\mathrm{beam}})$ was reduced to decrease the bending rigidity and increase structural compliance. For a rectangular beam, the area moment of inertia scales as $I\propto b{t_{\mathrm{beam}}}^{3}/12$ , and the maximum deflection ( $\delta_{max}$ ) under a given tip load ( F ) follows $\delta_{max}=FL^{3}/(3EI)\propto {t_{\mathrm{beam}}}^{-3}$ , indicating that the deflection increases strongly as the beam thickness decreases. A thinner cantilever can therefore yield a larger deformation and higher piezoelectric strain under the same aerodynamic loading. In addition, because the beam stiffness scales as $k\propto EI/L^{3}\propto {t_{\mathrm{beam}}}^{3}$ , reducing the thickness also contributes to lowering the natural frequency. Collectively, these two design modifications were intended to move the device toward a lower-frequency, more compliant operating regime with reduced reliance on resonance amplification.

A further design feature of the present harvester is the replacement of the conventional proof mass, typically attached to a silicon cantilever beam, with a cylindrical oscillator fabricated from polylactic acid (PLA), which is more suitable for flow-induced excitation. The MEMS energy harvester has overall dimensions of approximately 550 µm in width and length and 550 µm in height, and incorporates a cylindrical oscillator with a diameter of 2.00 mm and a height of 10.0 mm. Advanced micromachining techniques were used to integrate an aluminum nitride piezoelectric film onto the cantilever beam for electromechanical energy conversion. The voltage generated by the piezoelectric layer was extracted through aluminum electrodes connected to an external circuit with a 10 MΩ load resistance. In this configuration, the oscillator serves to transduce external fluidic forces into cantilever motion, thereby enabling electrical power generation from the resulting structural response.

Our energy harvester converts vibrational energy from the cantilever beam into electrical power via piezoelectric films. Upon application of external force, the cantilever beam undergoes alternating tensile and compressive stresses in the piezoelectric film, resulting in the generation of an oscillating electric potential. This generated voltage further influences the displacement of the piezoelectric film, forming a coupled mechanical–electrical system of equations. Notably, the finely tuned oscillator’s alignment with the flow conditions ensures continuous generation of Karman vortices, leading to the repetitive motion of the cantilever. This dynamic is encapsulated by Equations (1) and (2), highlighting the intricate interplay between the mechanical and electrical aspects of the harvester [33]:

(1) $$m\ddot{y}+c\dot{y}+ky+\Theta V=F_{L},$$

(2) $$C_{P}\dot{V}+\frac{V}{R}-\Theta \dot{y}=0,$$

where the overdot denotes differentiation with respect to time. The parameters *m*, *c*, *k*, *Θ*, *V*, *F_L_, C_p_*, and *R* represent the effective mass, damping coefficient, stiffness, electromechanical coupling coefficient, voltage, lift force, system capacitance, and electrical resistance, respectively. The electromechanical coupling coefficient represents the conversion efficiency between displacement and voltage. The lift force *F_L_*, generated by the Karman vortices around the oscillator is given by $F_{L}=C_{L}\rho u^{2}/2$ , where $C_{L}$ , ρ , and u are the lift coefficient, density of the fluid (air in this study), and flow velocity, respectively. Here, the flow around the oscillator undergoes changes due to the formation of Karman vortices, and it is these vortices that primarily influence the variations in $F_{L}$ [34,35]. This force initiates vibration of the oscillator, which in turn stimulates the cantilever beam’s motion, driving the energy harvesting process. From Equations (1) and (2), we can anticipate that $F_{L}$ has a significant impact on the oscillations of the cantilever beam (*y*) and the voltage (*V*) generated by the piezoelectric film. This underscores the importance of investigating the variations in $F_{L}$ due to the formation of Karman vortices when designing energy harvesters. We will explore the effect of $F_{L}$ around the oscillator through CFD analysis, followed by an experimental assessment of the voltage generated by the MEMS harvester.

### 2.2. Numerical Analysis of Fluid-Induced Forces on the MEMS Harvester

To accurately capture the fluid dynamics affecting our MEMS energy harvester, a computational analysis was performed, focusing on the influence of the Karman vortex on the oscillator. This detailed analysis is essential for refining the harvester’s design, particularly for optimizing its response to Karman vortex forces. Building upon previous research in fluid-induced vibration (FIV) [31,36], our study emphasizes the importance of lift force variations on the oscillator.

**Figure 2:**
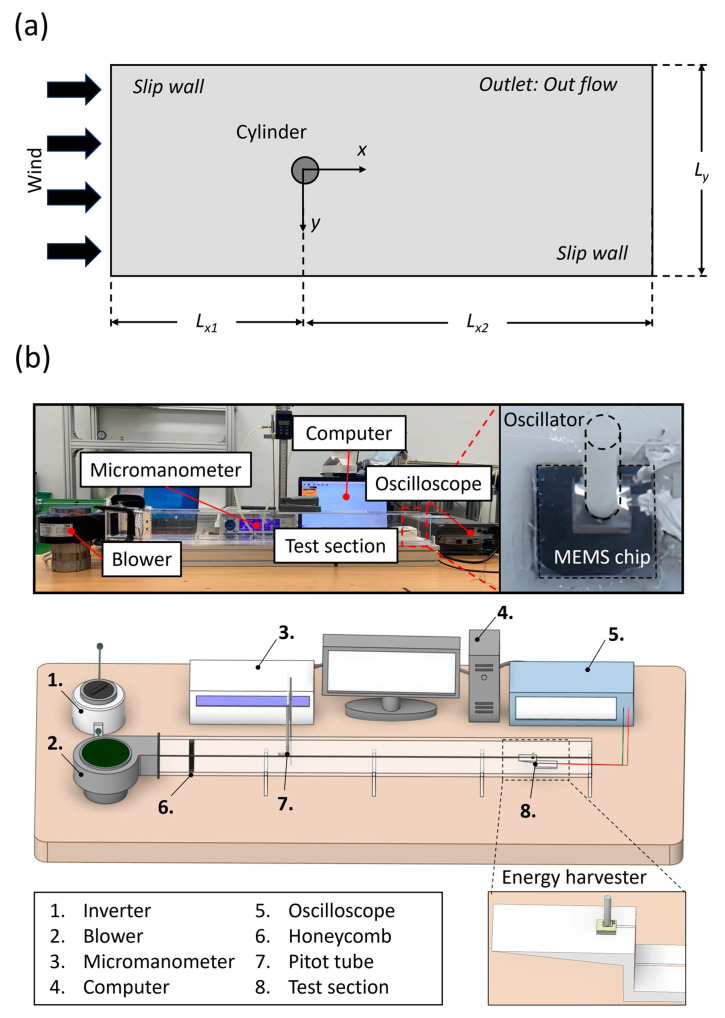
Aerodynamic force analysis and experimental setup: (**a**) Computational domain for aerodynamic force analysis of the cylinder. (**b**) Wind tunnel setup illustrating the arrangement of the blower, inverter, measurement instruments, and the test section for harvester performance evaluation.

The analysis domain was set similarly to emulate the phenomena observed in experiments. As illustrated in Figure 2a, the domain features a cylinder with a diameter (*D*) of 2.00 mm, identical to the oscillator of the energy harvester. The surrounding fluid flow field was defined by characteristic lengths L_x1_, L_x2_, and L_y_ of 50*D*, 150*D*, and 100*D*, respectively, chosen to minimize the influence of the lateral boundary conditions. For our numerical model, the foundation was set in two key governing equations: the mass conservation (continuity) equation and the momentum conservation equation. These equations offer crucial insights into the fluid behavior around the harvester. The mass conservation equation is given by

(3) $$\frac{\partial \rho }{\partial t}+\rho \nabla \cdot \vec{v}=0+\nabla \cdot \left(\rho \vec{v}\right)=0,$$

where *ρ*, *t*, and $\vec{v}$ are the air density, time, and velocity vector, respectively. This equation ensures that the mass is conserved in the flow field around the harvester. The momentum conservation equation is expressed as follows:

(4) $$\rho \frac{\partial }{\partial t}\left(\vec{v}\right)+\rho \nabla \left(\vec{v}\vec{v}\right)=-\nabla p+\nabla \cdot [\mu [\left(\nabla \vec{v}+{\vec{v}}^{T}\right)-\frac{2}{3}\cdot \nabla \vec{v}I]],$$

where ∇p , *μ*, ${\vec{v}}^{T}$ , and *I* are the pressure gradient, dynamic viscosity, transpose vector of velocity, and unit tensor, respectively. The boundary conditions for the CFD analysis were carefully chosen to reflect realistic physical interactions under the same conditions as the experiments. At the inlet, a uniform velocity condition was applied, while at the outlet, an outflow condition was set to simulate the fluid exiting the domain. The side surfaces were treated with symmetrical boundary conditions to mimic an infinite fluidic domain, thereby optimizing the computational efficiency and mirroring real-world conditions. The properties of the working fluid in the analysis were set to match the experimental environment, using air properties at atmospheric pressure and a standard temperature of 25.0 °C.

Using the commercial software ANSYS Fluent 2023 R1, we employed a discretization approach based on the finite volume method (FVM) to transform these continuous equations into a computationally manageable format. Second-order discretization schemes for velocity, pressure, and time variables were applied, ensuring higher accuracy. The SIMPLE algorithm was used for pressure–velocity coupling under laminar flow conditions (Re = 1095). A grid independence test was conducted to confirm that the chosen triangular mesh size accurately represented the fluid dynamics. The final mesh configuration consisted of approximately 58,000 nodes, providing a reliable balance between accuracy and computational efficiency. The time step was set to 2.00 × 10^−6^ s, selected through sensitivity analysis to fully capture the transient nature of the flow.

### 2.3. Experimental Setup for Evaluating Performance of MEMS Energy Harvester

Following our observations of the external forces applied to the oscillator through CFD analysis, we aimed to measure the voltage generated by the forces acting on the oscillator through experiments. To evaluate the efficiency of the MEMS-based energy harvester in capturing wind energy under controlled conditions, we designed a series of wind tunnel experiments.

The experimental setup, detailed in Figure 2b, comprised a wind tunnel with a constant cross-section of 71.0 mm × 63.0 mm and a length of 1000 mm. This configuration allowed for the establishment of fully developed flow conditions. The blower’s (Inno Tech, Changwon, Republic of Korea) speed was controlled using an inverter, and the airflow velocity was measured with a pitot tube. The test section, featuring a sharp leading edge, made via 3D printing (Cubicon, Seongnam-si, Republic of Korea), was placed at the center height of the wind tunnel to mitigate boundary layer effects. The experiments were conducted under controlled environmental conditions of atmospheric pressure and a standard temperature of 25.0 °C. We recorded the voltage generated by the harvester using an oscilloscope (Tektronix, Beaverton, OR, USA). 

## 3. Results and Discussion

In this section, we present the validation and implications of our MEMS energy harvester’s design and performance. We begin by examining the fluid dynamics that drive the device’s functionality and then detail the fabrication processes. Finally, we demonstrate how the device translates wind energy into usable electrical power, marking a significant advance in energy harvesting technology.

### 3.1. Characterization of Vortex-Induced Forces and Their Effect on MEMS Energy Harvester

**Figure 3:**
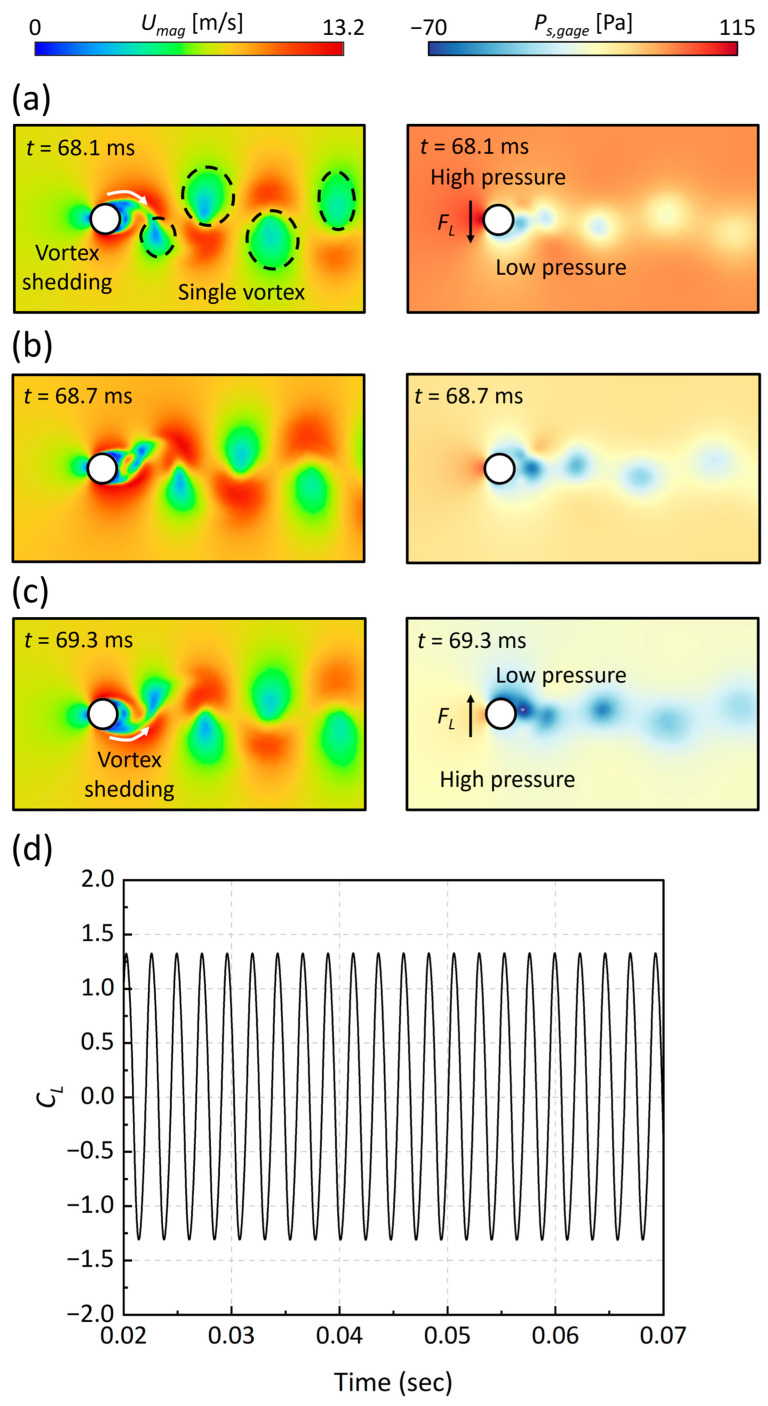
Fluid dynamics around the oscillator: (**a**–**c**) Transitional changes in the velocity and static pressure fields around an oscillator at an inlet velocity of 8.00 m/s (Re = 1095), depicting the influence on the lift force coefficient (*C_L_*). (**d**) Time domain signal of the *C_L_*, demonstrating its variation over time.

The CFD results of the transient changes in velocity (*U*) and static pressure (*P_s,gage_*) around the MEMS energy harvester, illustrating the lift coefficient (*C_L_*) evolution at an inlet velocity of 8.00 m/s, are presented in Figure 3. In this context, we analyzed the variation in the static pressure (*P_s_*) field, emphasizing that *P_s_* represents gauge pressure, due to its direct impact on the variation in the lift force caused by vortex formation. Between 68.1 ms and 69.3 ms, the formation of single-type vortices encircling the cylindrical oscillator was observed. The shedding of these vortices initiated at the oscillator’s circumferential boundary—referred to as the “top” in the 2D representation—and progressively migrated towards the “middle” and “bottom” regions in the 2D field. This migration led to significant alterations in the adjacent flow and pressure fields. At 68.1 ms, an elevated static pressure, reaching up to 115 Pa on the upper boundary in the 2D plane, generated a force directed towards the oscillator’s center. By 68.7 ms, a more uniform pressure distribution resulted in a neutral force impact on the oscillator. At 69.3 ms, the inversion of the pressure gradient, dropping to as low as −70 Pa, created a force that was directed away from the center. This sequence of dynamic shifts induced substantial lift forces, observable in Figure 3d, where the oscillator experienced periodic, sinusoidal forces, with the lift force coefficient peaking at 1.33.

Our CFD results elucidate that the vortex patterns that are proximal to the oscillator significantly manipulate the flow and pressure distribution, inducing periodic forces that are crucial for effective energy harvesting. These findings suggest that, within a flow environment, the MEMS energy harvester will prompt vertical oscillations in the silicon cantilever beam, which is critical for the harvester’s design to ensure optimal energy extraction.

### 3.2. Fabrication and Characterization of MEMS Energy Harvester and Piezoelectric Film

**Figure 4:**
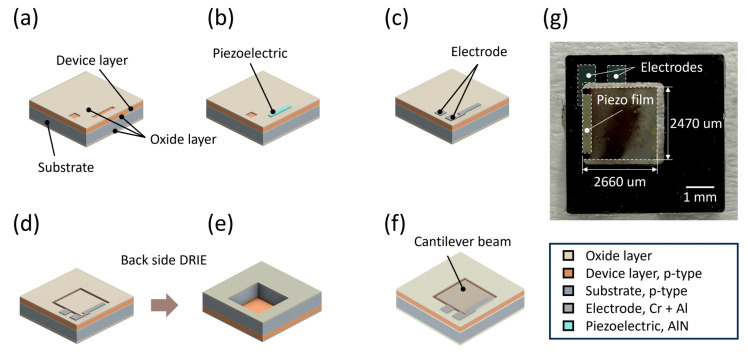
MEMS chip fabrication process: (**a**) SiO_2_ layer patterning for the piezoelectric AlN film deposition. (**b**) AlN film deposition. (**c**) Electrode layer deposition for electrical output. (**d**) Formation of the cantilever structure through reactive ion etching (RIE). (**e**) Finalization of the cantilever with back-side alignment and deep reactive ion etching (DRIE). (**f**) A schematic and (**g**) photograph of the completed MEMS chip with cantilever beam, piezoelectric film, and electrodes.

Building on the insights from Section 3.1, Figure 4 illustrates our fabrication process for the MEMS chip, tailored to incorporate the parameters from our vortex dynamics studies. The process begins with the cleaning of a 4-inch silicon-on-insulator (SOI) wafer, characterized by a 400 µm thick silicon handle layer, a 1.00 µm thick buried oxide layer, and a 5.00 µm thick p-type silicon device layer, selected for their superior electrical properties and versatility. To protect the device and handle layers during the fabrication and prevent edge oxidation, a protective SiO_2_ layer, approximately 3000 Å thick, was deposited on both surfaces of the wafer via plasma-enhanced chemical vapor deposition (PECVD).

For the piezoelectric component, aluminum nitride (AlN) was selected despite having a piezoelectric constant (5.5 × 10^−12^ C/N at d_33_) that is approximately 100 time lower than that of lead zirconate titanate (PZT) [37,38]. This choice was made considering AlN’s advantages, such as a reduced contamination risk and greater cost-efficiency [39]. AlN was sputtered to a 5000 Å thickness. The fabrication sequence involved patterning for piezoelectric layer deposition, etching, and a lift-off process to remove superfluous material, culminating in the construction of electrodes from layers of Al (3000 Å) and Cr (200 Å) through evaporation, essential for channeling the piezoelectric film’s electrical output. Here, in the construction process of the electrode, an electron beam (E-beam) evaporator was employed for depositing Al/Cr layers at thicknesses of 3000/200 Å. These layers were deposited atop the upper oxide layer and the sputtered AlN region to form the electrodes. Cr was used as an adhesive layer, which did not affect the AIN performance [40]. Forming a cantilever beam involved etching a specific pattern on the device layer, reactive ion etching down to the buried oxide layer, and the addition of a protective cap. This was followed by backside alignment, etching a masking pattern, and the use of deep reactive ion etching (DRIE) to sculpt the cantilever from the handle layer. The completed MEMS chip, as shown in Figure 4f,g, includes the cantilever beam, piezoelectric film, and electrodes.

**Figure 5:**
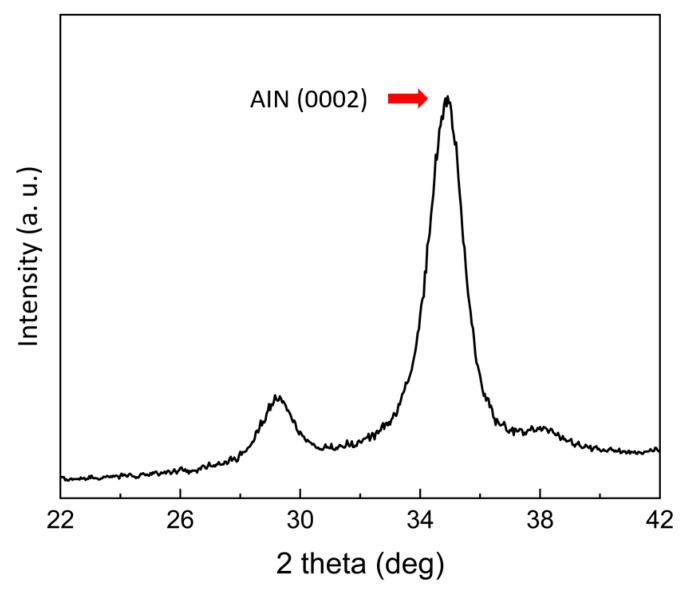
X-ray diffraction (XRD) analysis of the fabricated MEMS structure. Strong intensity peak at the 2*θ* position of around 36 degrees confirms the successful deposition of the AlN film in the (0002) orientation.

The thin AlN film, covered with electrodes and deposited on the silicon cantilever beam (Figure 4g), exhibits varying physical properties based on its crystal orientation. The reactive sputtering process, employed for depositing AlN, requires careful control of the sputtering pressure at 1 Pa and nitrogen gas concentration (50% N_2_/Ar) to ensure alignment in the (0002) direction [41]. X-ray diffraction (XRD) analysis of our fabricated structure confirmed the successful deposition of the AlN film in the correct orientation, as indicated by the strong intensity peak at the 2*θ* position of around 36 degrees in Figure 5, corroborating the perpendicular alignment of the film relative to the cantilever beam’s surface [41,42,43]. The final step in the device fabrication involved forming a cylindrical oscillator using a 3D printer. The oscillator made from polylactic acid filament was secured to the cantilever beam with a cyanoacrylate adhesive, measuring 2.00 mm in diameter and 10.0 mm in height.

### 3.3. Harnessing Wind Energy: Correlating Vortex-Induced Dynamics with Electrical Output

**Figure 6:**
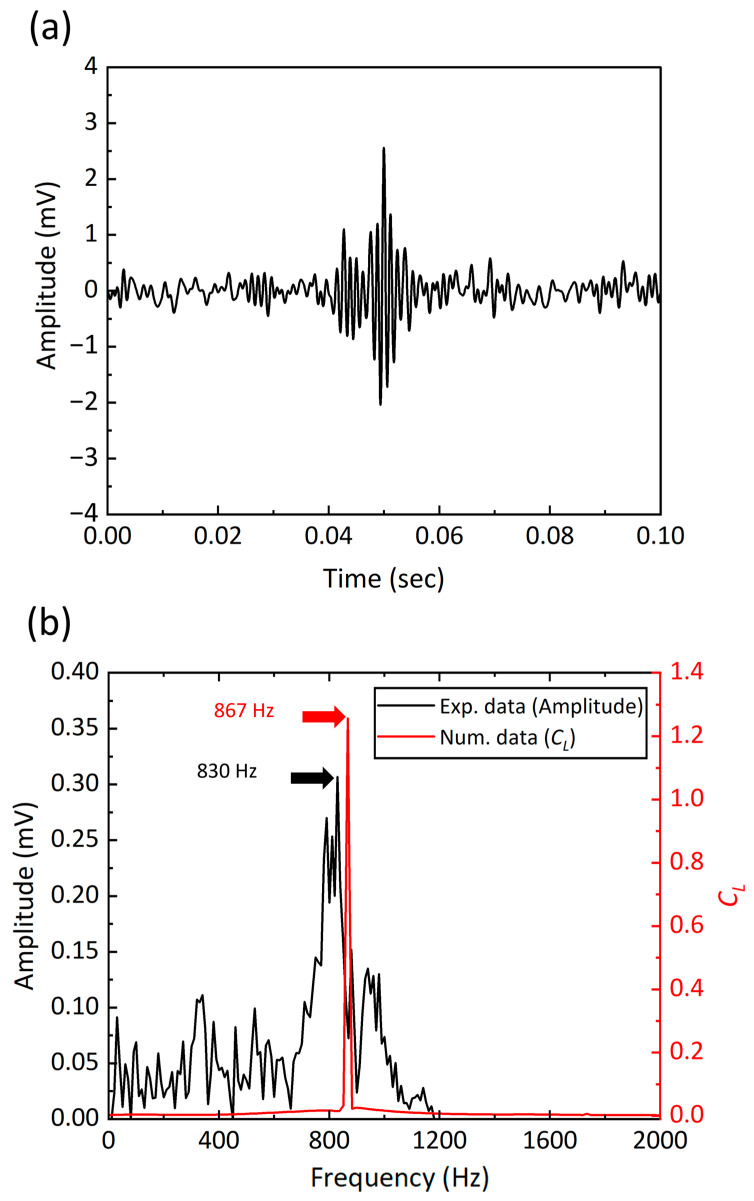
Output AC voltage signal and frequency analysis: (**a**) Time domain representation of the output AC voltage signal, showing variation and peak amplitude over time. (**b**) Frequency spectrum from FFT analysis, comparing experimental voltage with numerical simulation results for lift force coefficient (*C_L_*), indicating dominant frequencies at 830 Hz and 867 Hz.

This section investigates the harvester’s ability to harness wind energy and convert it into electrical energy. As evidenced by the generated AC voltage signals presented in Figure 6a, we observed distinct voltage signal generations across the time and frequency domains during the wind tunnel test. A notable peak at approximately 2.56 mV at around 50 ms is a clear indicator of the harvester’s ability to effectively capture wind energy. To ensure that this response is due to the changes in the vortex-induced lift force, we conducted a comparative analysis, correlating the experimental AC voltage signals with the lift force frequencies deduced from our numerical studies. Employing fast Fourier transform (FFT) analyses on both sets of data revealed dominant frequencies at 830 Hz and 867 Hz, as indicated in Figure 6b. The slight variance between the experimental frequency and the CFD-derived frequency points to minor discrepancies in the vortex formation around the MEMS chip, affecting the overall vortex shedding behavior.

The Strouhal number (*St*), a key dimensionless quantity in fluid dynamics, was assessed to validate the power signal’s reliability and consistency. This number, which ties the vortex shedding frequency to flow characteristics, is defined as follows [44]:

(5) $$St=\frac{fD}{u}$$

where *f* represents the vortex shedding frequency of the flow. In this context, it is essential to clarify that the “vortex shedding frequency” pertains exclusively to the frequency at which vortices are shed by the fluid flow, distinctly separate from the oscillation frequency of the structural. Considering the Reynolds number variations, the Strouhal number was experimentally determined to be approximately 0.21, resonating with the fluidic conditions and an inlet velocity of 8.00 m/s (Re = 1095) [44,45,46]. This led to a calculated vortex shedding frequency of around 840 Hz, closely aligning with the FFT analysis outcomes and affirming the influence of the vortex behavior on the electrical output of the harvester. 

We observed a voltage signal from the MEMS energy harvester of 2.56 mV at 830 Hz. By comparing the frequencies of vortex shedding, excitation (i.e., voltage), and lift force at 8 m/s, we demonstrated its applicability in actual flow environments. When designing piezoelectric MEMS energy harvesters, materials like PZT and AlN are considered. Although PZT is often favored for its superior piezoelectric efficiency [38], allowing vibrational energy harvesters to generate power ranging from 1 μW to 100 μW [16,17,18], it poses specific challenges in fabrication due to contamination risks during the deposition process. Conversely, while AlN exhibits lower piezoelectric properties, it is considered more feasible for MEMS fabrication due to its ease of processing [39], typically generating power at mV levels in the nW to μW range [19,20]. The relatively low power output from these harvesters presents challenges in electrical circuit functions, including rectification. Nonetheless, extensive research has been undertaken to enhance and convert power from the mV and nW-μW levels to scales that are practical for energy harvesting [21,22].

Methods to enhance performance include minimizing residual stresses on piezoelectric materials through precision micromachining processes and controlling Karman vortex patterns. In the process of harvesting wind energy, the AlN piezoelectric film, which is essential for converting mechanical energy into electrical energy, experiences changes in its coupling coefficient due to residual stresses induced during micromachining [47,48]. Increased residual stresses result in reduced coupling coefficients of the piezoelectric elements, subsequently impairing the performance of energy capture [48]. Therefore, addressing the challenges in micromachining to control or mitigate these residual stresses is crucial for enhancing the operational efficiency and energy harvesting capabilities of our MEMS energy harvester. Additionally, our computational fluid dynamics (CFD) studies reveal that a single vortex type predominates in the wake of the MEMS energy harvester, as depicted in Figure 3. By modifying the arrangement or the surface of the cylinder to promote the formation of combined single and pair of vortex patterns, we anticipate a possible increase in the amplitude of the voltage produced by the MEMS energy harvester [49].

## 4. Conclusions

In summary, we presented an MEMS energy harvester for capturing the flow-induced vibration caused by the Karman vortex. We designed and fabricated a 5 µm thick silicon cantilever beam coated with AlN piezoelectric film through MEMS processes. The alignment of the piezoelectric film was verified through an XRD test. The fluctuating static pressure field around the oscillator, induced by vortex formation, is the primary driver of the cantilever beam’s vibrations. The robust correlation between the wind-tunnel-derived voltage signals and the CFD lift force coefficients, especially regarding frequency alignment, as denoted by the Strouhal number, not only validates the harvester’s operational efficacy but also emphasizes the importance of flow field control in enhancing energy harvesting performance.

The ability of our energy harvester to transform wind-induced vibrations into electrical energy with substantial voltage outputs suggests the harvester’s practical applicability. A notable peak at approximately 2.56 mV at 830 Hz was a clear indicator of the harvester’s capability to effectively capture wind energy. The observed voltage generation is not just a measure of the device’s operational capability but also reflects its alignment with the anticipated performance. The dominant frequencies within the 830 Hz to 867 Hz range closely align with the theoretical analysis of vortex shedding dynamics. This agreement of experimental results with theoretical models solidifies our research methodology and supports the significance of precise modeling in the conceptualization of effective harvesting mechanisms. 

In sum, our findings not only elucidate the operational potential of MEMS energy harvesters but also lay the groundwork for subsequent innovation in the field of renewable energy technologies. Additionally, the suggested energy harvesting method is significantly influenced by fluid dynamics, the shape of the energy harvester, resonance phenomena, and structural characteristics. So, if optimal design is achieved in future works, it could lead to enhanced energy harvesting. The implications of our work possibly extend into the realm of sustainable energy solutions and low-power wireless sensors, where the application of MEMS technology could potentially play a transformative role.

## Data Availability

The original contributions presented in the study are included in the article, further inquiries can be directed to the corresponding author.
