# Correction: Kang et al. Fluid Flow to Electricity: Capturing Flow-Induced Vibrations with Micro-Electromechanical-System-Based Piezoelectric Energy Harvester. *Micromachines* 2024, *15*, 581

**DOI:** 10.3390/mi17060654

**Published:** 2026-05-26

**Authors:** Jin Gu Kang, Hyeukgyu Kim, Sangwoo Shin, Beom Seok Kim

**Affiliations:** 1 Department of Mechanical Engineering, Seoul National University of Science and Technology, Seoul 01811, Republic of Korea; nak9837@seoultech.ac.kr (J.G.K.); kimhyeuku@seoultech.ac.kr (H.K.); 2 Department of Mechanical and Aerospace, University at Buffalo, The State University of New York, Buffalo, NY 14260, USA; sangwoos@buffalo.edu; 3 Department of Mechanical and Automotive Engineering, Seoul National University of Science and Technology, Seoul 01811, Republic of Korea

With this correction, the Editorial Office together with the authors have made the following amendments to the published article [1]. 

**Figure Legend**

The legend for Figure 1 has been refined to explicitly highlight that the proposed device represents an extension of previously reported VIV-based configurations. In particular, the updated legend clarifies the design lineage by indicating that the present work builds upon and advances the framework reported in [29]. The correct legend appears below. 

**Error in Figure**

In the original publication, an issue was identified in Figure 1 as published. The schematic representation did not sufficiently distinguish the present device configuration from prior vortex-induced vibration (VIV)-based energy harvesting concepts, which may have led to ambiguity regarding the design novelty and intended high-Reynolds-number operating regime.

The corrected Figure 1 is provided below to clarify these distinctions. 

**Text Correction**

An issue was identified in the original publication concerning the clarity of the introductory text. The introductory paragraph did not sufficiently clarify that the present study does not seek to introduce the concept of vortex-induced vibration (VIV)-based energy harvesting, but rather to extend its applicability to MEMS devices operating under high-Reynolds-number, non-resonant flow conditions. In particular, the distinction between the present work and prior studies [Lee et al., Sci. Rep. 9, 20404 (2019)] could have been articulated more clearly, which may have led to potential ambiguity regarding the originality and scope of the proposed device. The relevant text has therefore been revised to explicitly state the design intent, namely, the development of a structurally reconfigured MEMS harvester optimized for high-Re sensitivity through reduced beam stiffness and increased oscillator mass to lower the resonant frequency, thereby enabling effective operation in dynamic, high-speed airflow environments beyond conventional resonance-based regimes. Corrections have been made to Section 1 (Introduction) and Section 2.1 (Design and Principle of MEMS Energy Harvester):

**- Revised paragraph in Section 1 (Introduction), Paragraph 4**

Building on these advances, flow-driven MEMS energy harvesting has also attracted growing attention. While vortex-induced-vibration-based harvesting concepts have been widely explored [28–32], most MEMS implementations have focused on resonance-based operation. In such designs, the response is maximized when the excitation frequency approaches the structural natural frequency (Figure 1b). However, resonance-based approaches inherently provide high performance only within a narrow frequency band, and the response can drop rapidly as operating conditions vary. Therefore, to achieve stable operation over a wide frequency range in realistic environments, a design principle that reduces reliance on resonance is needed. A representative application that demands wideband robustness is high-Reynolds-number flow. Using the Strouhal relation St=fD/U, the characteristic flow frequency is f=St U/D, indicating that for a fixed oscillator diameter D, f  increases with flow velocity (U). In the operating range considered here (U≈8 m/s, D≈2 mm), the device has a first natural frequency of approximately 78 Hz [29], whereas the characteristic flow frequency under high-speed conditions (U≥8 m/s) increases to approximately 840 Hz (Figure 1b), yielding a strong frequency mismatch ratio $f_{flow}/f_{n}\approx 10$. Under such conditions, resonance-tuned VIV operation becomes practically constrained.

**- Added paragraph in Section 1 (Introduction), Paragraph 5**

In this study, the device was redesigned to relax the dependence on strict resonance matching and to promote a more flow-governed harvesting response. As shown in Figure 1c, we adopted a cantilever–oscillator coupled configuration based on the cantilever–oscillator architecture [29]. In contrast to conventional designs that primarily rely on resonance matching, the present device was tailored for a high-Re operating condition in which the characteristic flow frequency is substantially higher than the structural natural frequency. To realize this operating regime, two structural modifications were introduced. First, the effective mass m of the oscillator was increased to reduce the natural frequency of the cantilever–oscillator system, following the scaling $f_{n}\propto \sqrt{k/m}$. Thus, increasing m shifts the structural resonance further below the working flow-induced excitation frequency. Second, the cantilever thickness ($t_{\mathrm{beam}}$) was reduced. For a rectangular cantilever, the area moment of inertia scales as $I\propto {t_{\mathrm{beam}}}^{3}$, and the maximum deflection under a given load follows $\delta_{max}=FL^{3}/(3EI)\propto {t_{\mathrm{beam}}}^{-3}$, indicating that the deflection increases strongly as the thickness decreases. In addition, since the bending stiffness scales as $k\propto EI/L^{3}\propto {t_{\mathrm{beam}}}^{3}$, reducing $t_{\mathrm{beam}}$ also lowers the natural frequency. Therefore, increasing m and reducing $t_{\mathrm{beam}}$ both contribute to lowering $f_{n}$, while the reduction in $t_{\mathrm{beam}}$ additionally promotes larger cantilever deflection under a given load. These two design features distinguish the present device from Ref. [29] and related prior work [28–32]. In the present design, the harvester is intentionally operated under a condition where the flow-induced excitation frequency is substantially higher than the structural natural frequency, while the thinner cantilever and heavier oscillator are used to enhance the beam deflection and further reduce $f_{n}$. To validate this concept, we employ a combined numerical and experimental approach. Two-dimensional computational fluid dynamics (CFD) simulations are performed to analyze vortex formation and fluid–structure interactions, while wind-tunnel experiments are conducted to characterize the resulting flow-induced response of the fabricated device. Through this integrated methodology, we assess the feasibility of the proposed architecture for energy harvesting under high-speed airflow conditions.

**- Revised paragraph in Section 2.1 (Design and Principle of MEMS Energy Harvester), Paragraph 1**

Our study presents a MEMS energy harvester intended for flow environments, extending beyond the conventional resonance-centered design framework of vibration-based harvesters. The device is based on the cantilever–oscillator architecture reported in Ref. [29], but was redesigned for operation under relatively high-speed flow conditions, where strict frequency matching becomes less practical. In this regime, the characteristic frequency of aerodynamic forcing increases with flow velocity and tends to deviate substantially from the structural natural frequency. Under such conditions, the harvester is required to produce sufficient structural strain even under off-resonant excitation. To address this requirement, two physics-based structural modifications were introduced. First, the effective oscillator mass was increased while maintaining the projected area of the oscillator. This was achieved by replacing the hollow cylindrical oscillator with a solid cylinder of the same outer diameter. In this way, the projected area was preserved, while the inertial loading of the oscillator was increased. From the scaling relation $f_{n}\propto \sqrt{k/m}$, an increase in the effective mass lowers the natural frequency of the cantilever–oscillator system. This modification was therefore intended to increase the frequency separation between the structural natural frequency ($f_{n})$ and the flow-induced excitation frequency ($f_{flow}$), thereby reducing the dependence of the device on strict resonance matching. Second, the cantilever thickness ($t_{\mathrm{beam}})$ was reduced to decrease the bending rigidity and increase structural compliance. For a rectangular beam, the area moment of inertia scales as $I\propto b{t_{\mathrm{beam}}}^{3}/12$, and the maximum deflection ($\delta_{max}$) under a given tip load (F) follows $\delta_{max}=FL^{3}/(3EI)\propto {t_{\mathrm{beam}}}^{-3}$, indicating that the deflection increases strongly as the beam thickness decreases. A thinner cantilever can therefore yield a larger deformation and higher piezoelectric strain under the same aerodynamic loading. In addition, because the beam stiffness scales as $k\propto EI/L^{3}\propto {t_{\mathrm{beam}}}^{3}$, reducing the thickness also contributes to lowering the natural frequency. Collectively, these two design modifications were intended to move the device toward a lower-frequency, more compliant operating regime with reduced reliance on resonance amplification.

**- Added paragraph in Section 2.1 (Design and Principle of MEMS Energy Harvester), Paragraph 2**

A further design feature of the present harvester is the replacement of the conventional proof mass, typically attached to a silicon cantilever beam, with a cylindrical oscillator fabricated from polylactic acid (PLA), which is more suitable for flow-induced excitation. The MEMS energy harvester has overall dimensions of approximately 550 µm in width and length and 550 µm in height, and incorporates a cylindrical oscillator with a diameter of 2.00 mm and a height of 10.0 mm. Advanced micromachining techniques were used to integrate an aluminum nitride piezoelectric film onto the cantilever beam for electromechanical energy conversion. The voltage generated by the piezoelectric layer was extracted through aluminum electrodes connected to an external circuit with a 10 MΩ load resistance. In this configuration, the oscillator serves to transduce external fluidic forces into cantilever motion, thereby enabling electrical power generation from the resulting structural response.

The authors state that the scientific conclusions are unaffected. This correction was approved by the Academic Editor. The original publication has also been updated.

## Figures and Tables

**Figure 1:**
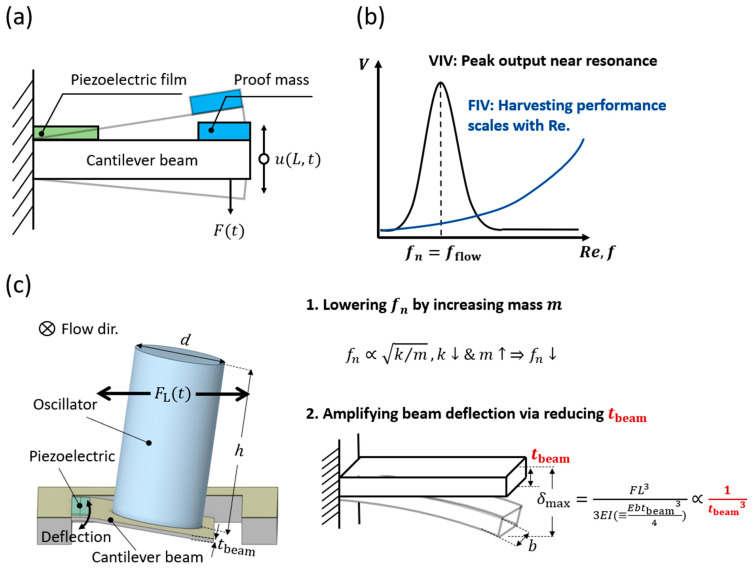
Schematics of energy harvesters: (**a**) Traditional MEMS-based vibration harvester consisting of a piezoelectric cantilever beam and a proof mass. (**b**) Conceptual comparison of operating regimes, where VIV-based harvesting peaks near resonance, whereas FIV-based harvesting seeks a sustained response as the Reynolds number increases. (**c**) Redesigned cantilever–oscillator harvester based on [29], retuned for high-Reynolds-number, off-resonant operation. The oscillator is subjected to an aerodynamic lift force acting perpendicular to the flow direction, which serves as primary time-varying excitation source for vibration. Increasing the oscillator mass m lowers the natural frequency $f_{n}$, shifting it further below the flow-induced excitation frequency (upper-right inset). Reducing the beam thickness (t)  lowers the bending rigidity $\left(EI\right)$, resulting in a larger deflection $\left(\delta \propto {t_{\mathrm{beam}}}^{-3}\right)$ and enhanced piezoelectric strain (lower-right inset).
